# Appraisal of the Effectiveness of Pelvic Proprioceptive Neuromuscular Facilitation Methods to Optimize Balance and Gait in an Eight-Year-Old Child

**DOI:** 10.7759/cureus.29648

**Published:** 2022-09-27

**Authors:** Vikrant G Salphale, Rakesh K Kovela, Moh'd Irshad Qureshi

**Affiliations:** 1 Department of Neuro Physiotherapy, Ravi Nair Physiotherapy College, Datta Meghe Institute of Medical Sciences, Wardha, IND; 2 Department of Physiotherapy, Nitte Institute of Physiotherapy, Nitte (Deemed to be University), Mangalore, IND

**Keywords:** task-oriented training, pelvic malalignment, gait parameters, pelvic proprioceptive neuromuscular facilitation, spastic diplegia

## Abstract

Among the different variants of cerebral palsy (CP), spastic diplegia has a greater frequency than the other variants, with each subtype having a diverse clinical presentation. Pelvic asymmetry is observed frequently in children with spastic diplegia which influences the functional abilities of the child such as balancing abilities and independent walking. Currently, physical therapists tackle this condition through numerous strategies of treatment, with each treatment strategy having its own significance. This case report emphasizes the effectiveness of pelvic proprioceptive neuromuscular facilitation (PNF) techniques in optimizing the balance and gait parameters in an eight-year-old female child who presented with spastic diplegia. The child came into the outpatient Department of Neuro Physiotherapy with complaints of delay in attaining milestones according to her age as well as her inability to balance and walk on her toes. History revealed that there was a delayed cry pointing toward birth asphyxia and the linkage of birth asphyxia in the emergence of CP. Pelvic PNF has a beneficial impact on optimizing trunk control and stability. This research presents evidence that pelvic PNF optimizes the balancing capacities and gait parameters and rectifies the malalignment of the pelvis in children with spastic diplegia. The findings of this case report prove that pelvic malalignment which influences the balance and walking abilities of the child can be rectified and tackled with pelvic PNF techniques.

## Introduction

Developmental delays are common in children who are delivered before the end of the pregnancy. The phrase developmental delay refers to any delay in a child in meeting milestones appropriate for age [1]. The developmental rules suggest that the entire process of development proceeds in a cephalo-caudal manner, implying that neck control is followed by trunk control, trunk control is followed by pelvic control, and pelvic control is followed by the control and stability of lower limbs. Milestones reflect the abilities that a child attains following age progression [2].

Cerebral palsy (CP) is a collection of lifelong movement and posture issues that limit activity and are thought to be caused by non-progressive disturbances in a developing fetal or infant brain [3]. It is a neurodevelopmental condition with stagnation that manifests as various musculoskeletal deformities, such as interference with pelvic geometry and spine alignment [4]. Such children additionally show a compromise in their cognitive and particular senses in addition to these issues. These issues limit the ability of these kids to complete any activity because both the neurological and musculoskeletal systems need to be in sync for any task to be completed.

The causes of CP are innumerable and have been divided into three categories, namely, prenatal, neonatal, and postnatal. Neonatal causes are those that can harm the fetus immediately after birth, while postnatal causes include those that can delay the achievement of developmental milestones after birth. Antenatal factors include those that can stop the development of the fetus before birth, neonatal factors are sub-categorized as those that can harm the fetus at birth, and postnatal factors occur after birth [5]. The prevalence of CP varies according to geographic region and ranges from two to three infants per 1,000 live births. Prematurity is one of the factors that might cause CP to arise. The most common type of CP is spastic diplegia, one of several types [6].

The brain receives about 800 mL of blood per second through its vascular systems, and three minutes is the ideal amount of time for the brain’s tissues to withstand anoxia. The brain sustains irreparable damage if the dietary need is not met in three minutes [7]. Therefore, birth asphyxia is a frequent cause for the origin of CP. Congenital anomalies have a rare identification.

The diagnosis of CP is clinical and solely depends on a thorough evaluation by professionals. Therefore, collecting a child’s medical history is essential for confirming a diagnosis in such cases. In general history, the relationship between the factors that gave rise to the issue is of the greatest importance. Due to the wide spectrum of difficulties present in such children, the treatment of CP necessitates a multi-centered strategy, which calls for the involvement of specialists in various fields to carry out their respective duties [8]. Many different treatment philosophies and methods have been developed recently, and a physical therapist who works with CP patients uses and implements them. This case study demonstrates the effectiveness of pelvic proprioceptive neuromuscular facilitation (PNF) methods in correcting pelvic misalignment, thereby assisting in improving gait and balance outcomes in an eight-year-old female child.

## Case presentation

Patient information

An eight-year-old female child reported to the outpatient Department of Neuro Physiotherapy. The mother of the child provided complaints of lag in the attainment of milestones according to the age of the child. While recording the history to correlate the events responsible for spastic diplegia, we came to know there was a delay in the cry which pointed toward birth asphyxia. The family history taken from the mother of the child revealed that there was no consanguinity among the parents. Along with the family history, the mother reported that the child stayed in the neonatal intensive care unit for six days, and the immunization of the child was completed as per the age. On observation, the gross motor abilities of the child were favoring her to be included in the third level of the Gross Motor Function Classification System (GMFCS).

Clinical findings

**Figure 1:**
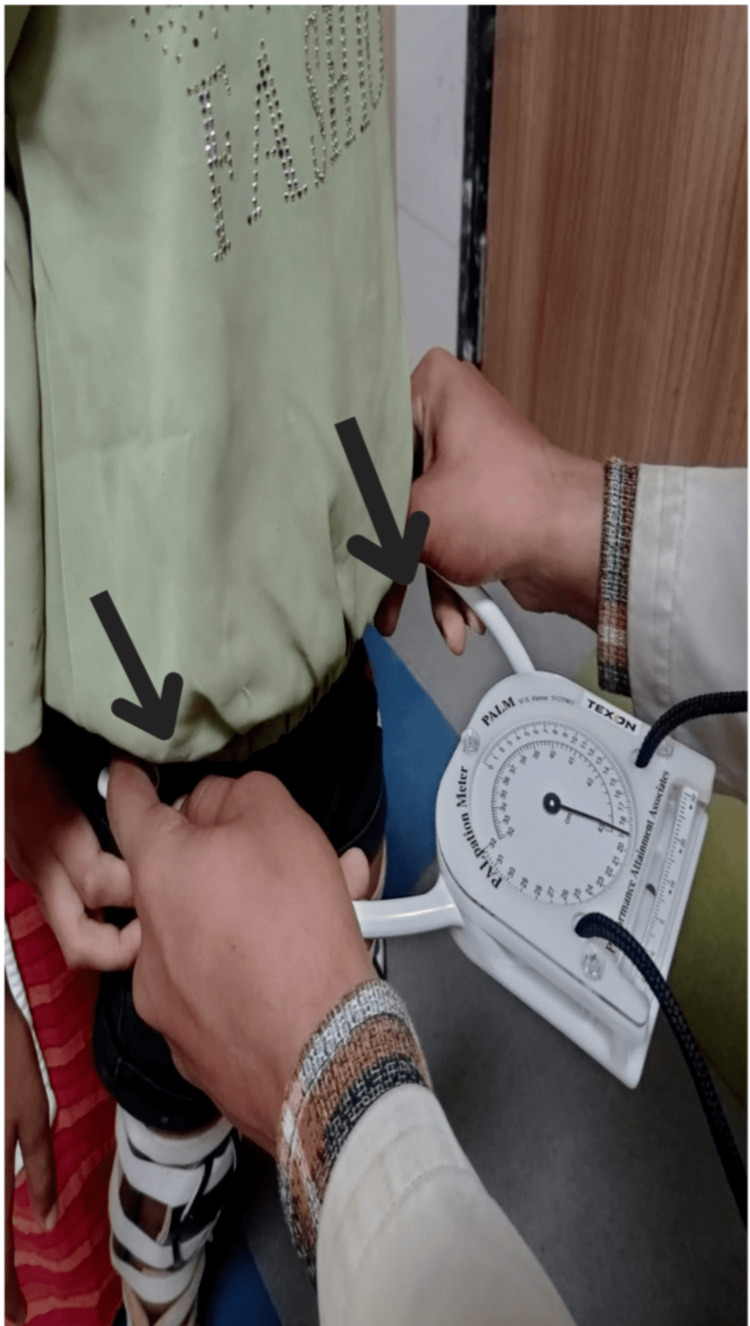
Assessment of the extent of asymmetry in the pelvis using the palpation meter device.

Observational findings of the child did not show any malalignment in the upper extremities, both shoulders were symmetrical, and the neck was in anatomical position. In contrast to the upper extremities, the lower extremities showed malalignment in the pelvis which was quantified by a palpation meter device (PALM). The baseline measurement on PALM showed a discrepancy of 0.7 inches in the pelvis of the dominant side (Table 1, Figure 1). Motor examination revealed the presence of spasticity in both lower limbs with exaggerated tendon jerks, and the baseline score on the pediatric balance scale was 46.

**Table 1:** Pre- and post-treatment findings of the patient gathered by pre- and post-treatment assessment.

Outcome measures	Baseline scores	Post-treatment scores
Pediatric balance scale (PBS)	46/56	52/56
Palpation meter readings (PALM)	0.7 inches	0.3 inches
Cadence (steps/minute)	78 steps/minute	96 steps/minute
Stride length (cm)	44 cm	60 cm
Gait velocity (m/s)	0.4 m/s	0.8 m/s

Plan of treatment

Goals of Treatment

Rectification of pelvic malalignment and optimization of balancing and walking abilities in the child.

Strategies of Treatment

The child received rhythmic initiation and slow reversal techniques of pelvic PNF for 15 minutes on both sides and the session was delivered six days a week. The same sequence was followed for four weeks. The performance of rhythmic initiation was initiated from the passive range of motion by following the principles of PNF. The passive range was followed by active assisted, active, and, at the termination, active resisted movement was followed. Rhythmic initiation is known to enhance strength and stability. On the other hand, the performance of slow reversals involved an isotonic contraction of prime movers, followed by a similar contracting mechanism of the antagonists.

**Figure 2:**
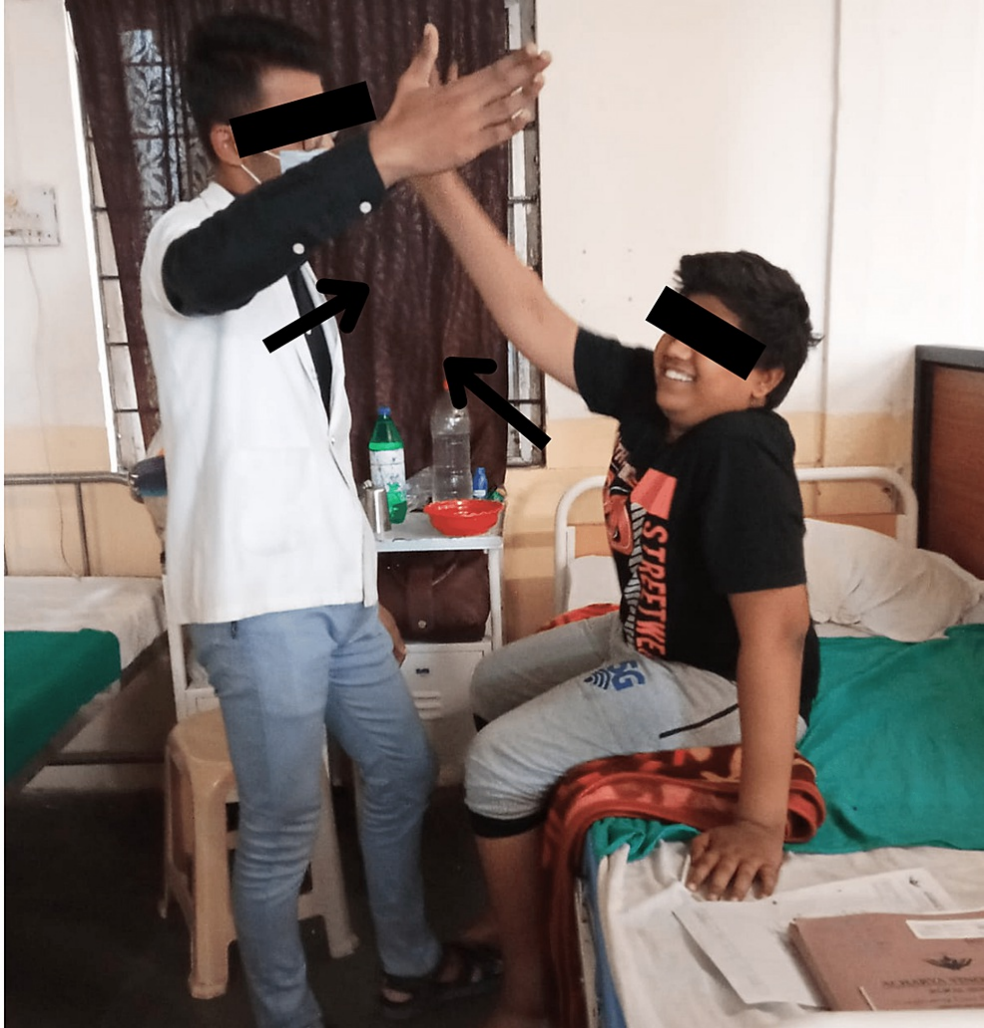
Delivering reach-out activities to the child.

Task-oriented exercises involved the performance of activities with the main emphasis on a particular target wherein the child was asked to achieve a particular target through the exercises. The exercises were reaching-out activities and walking on the ground. Reaching activities were performed in different fundamental positions where the child was asked to reach up to the hand of the therapist which was placed at a particular distance, and while reaching up to the hand of the therapist, the child had to modify her base of support which altered her center of gravity position (Figure 2). The base of support is a minimal or threshold area on which the body stabilizes itself to maintain a state of equilibrium, and if the base is wider, the center of gravity will be in a low position which means the stability would be at its optimum level. Usually, the position of the center of gravity is anterior to the second sacral vertebra. Thereby, reach-out activities enhanced the dynamic balance of the child. Walking on the ground or floor was beneficial in optimizing the gait parameters. Task-oriented training was given for half an hour and for six days a week which was followed up for four weeks.

## Discussion

The aim of this report was to investigate the impact of pelvic PNF techniques on balance, gait parameters, and in the rectification of pelvic malalignment in a child suffering from spastic diplegia. Musculoskeletal abnormalities, mostly pelvic malalignment, are quite prevalent in spastic diplegia patients which influence the functional abilities of the child such as independent transitions and walking [9]. Optimal performance of a particular movement requires coordination between the neural and musculoskeletal systems; therefore, to restore functional competencies in such children, the rectification of musculoskeletal issues is crucial for a therapist. Previous literature has provided strong evidence regarding the effect of pelvic PNF on the promotion of trunk control [10]. According to previous research, task-oriented training is an effective treatment strategy spastic diplegia patients [11]. Based on previous studies, a hypothesis was framed that pelvic PNF techniques will prove to be an effective treatment strategy in the rectification of pelvic malalignment, thereby influencing balance and gait parameters. According to the findings, a marked improvement was noticed in all the outcomes of group A in which the subjects received pelvic PNF techniques along with task-oriented exercises compared with group B which received only task-oriented exercises [11].

## Conclusions

Through this report, we concluded that pelvic PNF is an effective treatment strategy to enhance the balance and gait parameters as well as to regain pelvic alignment in a child with spastic diplegia. A majority of children with spastic diplegia present with a malalignment of the pelvis which interferes with the gross motor functioning or gross motor efficiency of such children. Therefore, according to the findings of this report, pelvic PNF is an effective treatment strategy to regain normal pelvic congruency as well as the gross motor efficiency of children with spastic diplegia.
